# The safety and efficacy of Sclerosing foam on treating venous leg ulcers: Protocol for systematic review and meta-analysis

**DOI:** 10.1097/MD.0000000000021608

**Published:** 2020-08-07

**Authors:** Weijing Fan, Xiao Yang, Baozhong Yang, Changgeng Fu, Renyan Huang, Feng Xv, Guobin Liu

**Affiliations:** a Shuguang Hospital Affiliated to Shanghai University of Traditional Chinese Medicine; b Shanghai University of Traditional Chinese Medicine, Shanghai; c Dongfang Hospital Affiliated to Beijing University of Chinese Medicine, Beijing, China.

**Keywords:** effect, safety, Sclerosing Foam, venous leg ulcers

## Abstract

**Background:**

Venous leg ulcers (VLUs) are common throughout the world, which seriously affects the patient's work and life. Relevant researches suggested that sclerosing foam (SF) has potential benefits for VLUs. However, there is no consistent conclusion. The purpose of our study is to assess whether SF is effective and safe for VLUs.

**Methods:**

Relevant clinical randomized controlled trials will be obtained from a search of 8 databases (with no language restrictions) from their inception to May 2020: PubMed, the Cochrane Library, EMBASE, Web of Science, China National Knowledge Infrastructure Database, Wanfang Database, China Science and Technology Journal Database, and Chinese Biological Medicine. Data will be analyzed using RevMan 5.3 after literature screening and data extraction according to predefined inclusion and exclusion criteria. Cochrane Collaboration Risk of bias Tool will be applied in evaluating the quality of enrolled articles. The primary outcome is Closure of venous leg ulcers, ulcer healing rate, adverse events related to SF. The secondary outcomes include ulcer healing time, ulcer recurrence rate, pain. Risk ratio will be used for categorical data; mean differences will be used for measurement data. Where possible and appropriate, meta-analysis will be performed for each outcome.

**Results:**

To clarify whether Sclerosing foam can be safe and efficient on treating venous leg ulcers.

**Conclusion:**

Our review will provide useful information to judge whether Sclerosing Foam is an effective and safe intervention for patients with venous leg ulcers.

## 1 Introduction

Venous leg ulcers (VLUs) are known as the most severe presentation of venous insufficiency that usually occur in the boot area (from below the ankle to the middle of the leg, mainly on the inside).^[1]^ Venous hypertension is the main cause of the disease.^[2]^ Long healing time, high care cost, and high recurrence rate are the main reasons making it a major health problem. Its prevalence in the United States is approximately 10% to 35%, ^[3]^ The general treatment options on VLUs include compression therapy, hyperbaric oxygen therapy, surgical debriefing, skin grafting, growth factors, and so on.^[4–6]^ However, venous hypertension still cannot be solved successfully. VLUs were estimated to cost US healthcare payers $14.9 billion every year.^[7]^

Compression is the cornerstone for treatment of venous ulcers but does not treat the underlying cause of venous hypertension^[6]^. Endovenous Ablation has proven its efficacy in the treatment of venous ulcers.^[5]^ However, Endovenous Ablation is not possible in every patient, for example, reflux in the distal great saphenous vein or tributaries. Therefore sclerotherapy is a very nice and good option.

Sclerosing Foam (SF) is a therapeutic method that uses liquid hardening drugs to cause a sterile inflammatory reaction of the venous wall, resulting in venous formation of fibrous cord, which achieves the overall purpose of treating venous hypertension.^[8]^ SF is accepted as a safe and effective treatment for venous insufficiency and especially for various venous diseases caused by venous hypertension.^[9–13]^ Emerging evidence of several randomized controlled trials (RCTs) published in recent years suggests a benefit of SF in patients with VLUs.^[14–16]^ A prospective randomized study from Kulkarni et al^[17]^ shows that SF agents can significantly improve the healing rate of VLUs and reduce the recurrence rate of VLUs. Another study showed that correction of superficial venous reflux in the lower limbs can help the healing of venous ulcers in the lower limbs.^[18]^

Despite many benefits for the treatment of venous disease, the safety of SF has been a concern. Reports show that 3.2% of patients develop deep vein thrombosis after sclerotherapy, and phlebitis occurs in 4.7% of patients after sclerotherapy.^[19]^ Therefore, whether they are effective and safe for VLUs remains to be assessed through systematic review and meta-analysis. The aim of this systematic review is to assess the efficiency and safety of SF for adults with VLUs.

## 2 Methods

### 2.1 Inclusion criteria for study selection

#### 2.1.1 Types of studies

Only RCTs about Sclerosing Foam on treating venous leg ulcers will be included, nonrandomized trials and observational studies will be excluded. There will be no restrictions on publication date and language.

#### 2.1.2 Types of patients

The adult patients (aged 18 years or older) who have been confirmedly diagnosed with VLUs. The Diagnostic Criteria for VLUs: Refer to the Evidence-based (S3) guidelines for diagnostics and treatment of venous leg ulcers.^[20]^

#### 2.1.3 Types of interventions

We will include studies that use any type of SF intervention, alone or as an adjunct to standard medical care for VLUs (liquid sclerotherapy will be excluded). We will compare this to a control group that receives standard medical care alone (without SF), or a control intervention other than SF.

#### 2.1.4 Types of outcome measures

The time period from start of treatment until the time point of outcome assessment will be defined as within 6 months. The time period from end of treatment until end of follow-up will be defined as within 1 year.

Primary outcomes

Closure of venous leg ulcers

Ulcer healing rate

Adverse events related to SF (such as phlebitis, deep vein thrombosis)

Secondary outcomes

Ulcer healing time

Ulcer recurrence rate

Pain related to VLUs (measured using any validated scales, such as verbal rating scale, or Visual Analogue Scale.

### 2.2 Search methods for the identification of studies

#### 2.2.1 Electronics searches

Eight databases will be searched from inception to May 2020: PubMed, the Cochrane Library, EMBASE, Web of Science, China National Knowledge Infrastructure Database, Wanfang Database, China Science and Technology Journal Database, and Chinese Biological Medicine. There are no article language restrictions.

The following search terms will include: “venous leg ulcers,” “venous leg ulcer,” “venous ulcer,” “Varicose Ulcer,” “Venous Hypertension Ulcer,” “Venous Stasis Ulcer,” “Venous Stasis Ulcers,” “Sclerosing Foam,” “Sclerotherapy,” “Foam Sclerotherapy,” “Sclerosing Solutions,” “Randomized controlled trial,” “Controlled clinical trial,” “clinical trial,” “ trial.” The search strategy for PubMed is shown in Table 1.

**Table 1:**
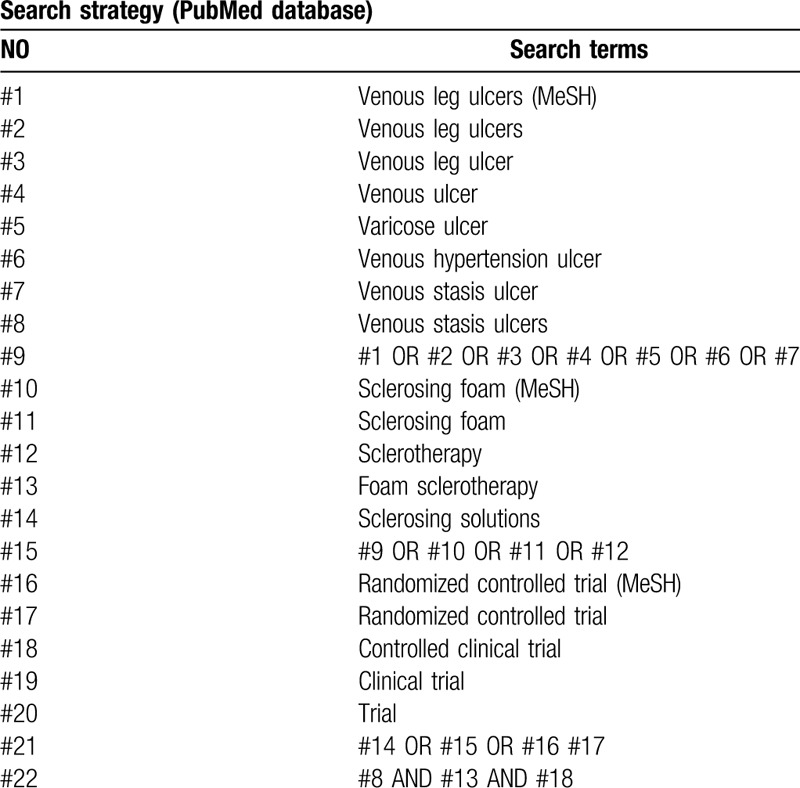
Search strategy used in PubMed database.

#### 2.2.2 Searching other resources

We will search the following trials registries: ClinicalTrials.gov, World Health Organization International Clinical Trials Registry Platform.

We will also review the reference lists of all major studies, and review the included studies for additional references. We will contact relevant experts to identify any unpublished research, or publications of a study in nonindexed journals.

### 2.3 Data collection and analysis

#### 2.3.1 Selection of studies

**Figure 1:**
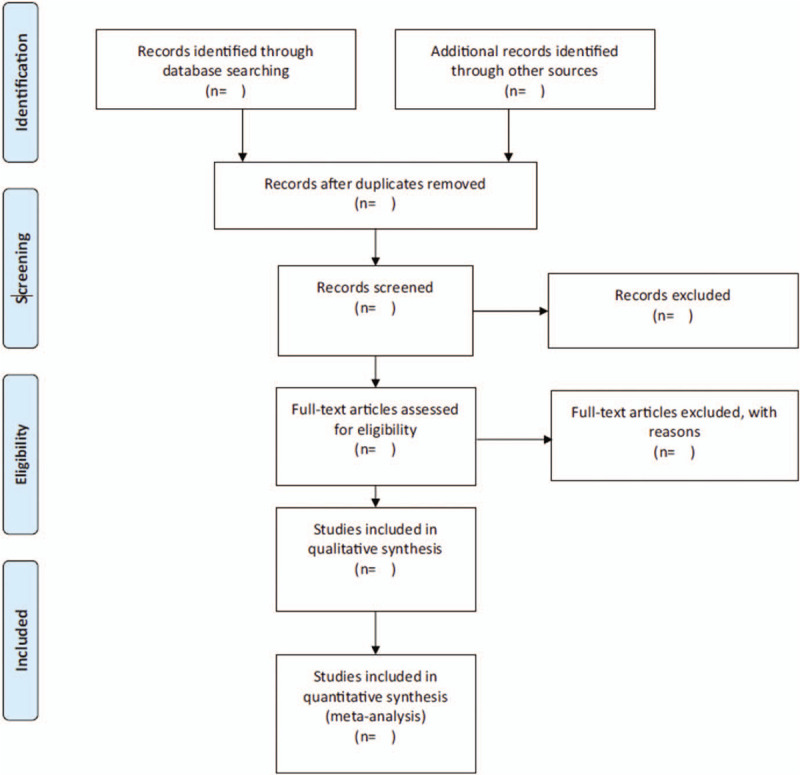
The PRISMA flow chart.

Two researchers (WF, CF) will independently screen the titles and abstracts identified by searches. If there is any disagreement in screening decisions, a third author (XY) will arbitrate. Later, they will independently screen the full texts to identify those studies that meet the inclusion criteria, and will record the reasons for the exclusion of ineligible studies. The different opinions will be resolved by discussions or consultation with a third reviewer (BY). The final selection flowchart follows the PRISMA guidelines, as shown in Figure 1.^[21]^

#### 2.3.2 Data extraction and management

Data extraction will include author names, publication date, study samples, interventional measures for the experimental and control treatments, efficacy evaluation indicators, treatment course, follow-up duration, randomization methods, baseline equilibrium, blinding, adverse effects reports, and results. The eligible studies will be screened by 2 reviewers independently based on previously determined criteria. Summary of the included studies will be shown in Table 2.

**Table 2:**
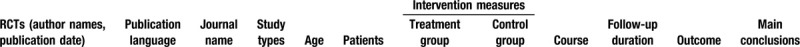
Summary of the included studies.

#### 2.3.3 Assessment of risk of bias in included studies

According to the Cochrane Handbook standards,^[22]^ the evaluation will include the following 7 aspects: random sequence generation (selection bias); distribution data hiding (selection bias); blinding (implementation bias) of researchers and implementers; results of the blind evaluation (measurement bias); completeness of outcome data (follow-up bias); results of selective reporting (reporting bias); and other sources of bias. Each item will be divided into 3 risk levels (low risk, unclear risk, high risk). Two researchers will independently evaluate the included literature and any disagreement will be resolved by consultation with a third party.

#### 2.3.4 Measures of treatment effect

Data analysis will be carried out using RevMan 5.3 provided by the Cochrane Collaboration. Risk ratio will be used for categorical data; mean differences will be used for measurement data, and 95% confidence intervals will be used.

#### 2.3.5 Dealing with missing data

We will record missing and unclear data for each included study. If possible, we will contact original authors to request missing data if necessary.

#### 2.3.6 Assessment of heterogeneity

Heterogeneity will be assessed using the I^2^ statistic. If I^2^ is not greater than 50%, which indicates no heterogeneity or slight heterogeneity, a fixed effects model will be used to combine statistical effects. If I^2^ is more than 50%, which indicates moderately severe heterogeneity, a random effects model will be used to combine the statistical effects. Subgroup analysis will be conducted according to the source of heterogeneity.

#### 2.3.7 Assessment of reporting bias

Reporting bias will be identified using funnel plot analysis; an even distribution of studies on either side of the vertical line of the combined OR value indicates no reporting bias.

#### 2.3.8 Subgroup analysis

We plan to carry out the following subgroup analyses if there are adequate studies:

Dose of SF (diFerent drug concentrations and frequencies)

Period of follow-up

Geographical area

We will use the formal test for subgroup interactions in Review Manager.^[23]^

#### 2.3.9 Sensitivity analysis

We will use sensitivity analysis to determine whether our results are robust. We will exclude the studies with high risk for bias from the summary analysis and analyze them again to assess the impact of these studies on the results.

#### 2.3.10 Grading the quality of evidence

The Grading of Recommendations Assessment, Development, and Evaluation guidelines^[24]^ will be applied to evaluate the quality of evidence of the including studies in our review from 5 considerations, included limitation of study design, inconsistency, indirectness, imprecision, and bias of publication. Additionally, the levels of evidence quality will be classified into 4 levels: “very low,” “low,” “moderate,” or “high” judgment.

## 3 Discussion

VLUs is one of the most common venous diseases in approximately one-third of the Western population, and its prevalence is increasing every year, it brings a lot of trouble to patients’ daily life. However, the effective treatment for VLUs is still controversial. ^[25]^ As a mature treatment to correct venous reflux and improve venous hypertension in the lower limbs, SF has shown great potential in the treatment of VLUs. However, there is lack of data on sclerosing foam on treating venous leg ulcers using evidence-based medicine. We believe this research will help vascular surgeons make decisions about whether or not SF can be used as an adjunct to VLUs treatment.

## Acknowledgment

The authors thank Diane Williams, PhD, from Liwen Bianji, Edanz Group China (www.liwenbianji.cn/ac) for editing the English text of a draft of this manuscript.

## Author contributions

**Conceptualization:** Weijing Fan, Guobin Liu.

**Data curation:** Xiao Yang, Renyan Huang, Feng Xv.

**Formal analysis:** Weijing Fan, Baozhong Yang, Changgeng Fu.

**Funding acquisition:** Guobin Liu.

**Investigation:** Xiao Yang, Renyan Huang, Feng Xv.

**Methodology:** Weijing Fan, Guobin Liu.

**Project administration:** Weijing Fan, Baozhong Yang, Changgeng Fu.

**Resources:** Weijing Fan, Guobin Liu.

**Software:** Weijing Fan.

**Supervision:** Baozhong Yang, Changgeng Fu.

**Validation:** Xiao Yang, Renyan Huang, Feng Xv.

**Visualization:** Xiao Yang, Renyan Huang, Feng Xv.

**Writing – original draft:** Weijing Fan.

**Writing – review & editing:** Weijing Fan, Guobin Liu.
